# The RNA-binding protein ESRP1 promotes human colorectal cancer progression

**DOI:** 10.18632/oncotarget.14318

**Published:** 2016-12-28

**Authors:** Sharmila Fagoonee, Gabriele Picco, Francesca Orso, Arrigo Arrigoni, Dario L. Longo, Marco Forni, Irene Scarfò, Adele Cassenti, Roberto Piva, Paola Cassoni, Lorenzo Silengo, Emanuela Tolosano, Silvio Aime, Daniela Taverna, Pier Paolo Pandolfi, Mara Brancaccio, Enzo Medico, Fiorella Altruda

**Affiliations:** ^1^ Institute of Biostructure and Bioimaging, CNR, Department of Molecular Biotechnology and Health Sciences, University of Turin, Italy; ^2^ Molecular Biotechnology Center, Department of Molecular Biotechnology and Health Sciences, University of Turin, Italy; ^3^ Candiolo Cancer Institute-IRCCS, University of Turin, Italy; ^4^ S.C. Gastroenterologia U, Endoscopia San Giovanni A.S., Azienda Città della Salute e della Scienza di Torino, Turin, Italy; ^5^ EuroClone S.p.A Research Laboratory, Molecular Biotechnology Centre, University of Turin, Italy; ^6^ Center for Experimental Research and Medical Studies, University of Turin, Italy; ^7^ Department of Medical Sciences, University of Turin, Italy; ^8^ Cancer Research Institute, BIDMC, Harvard Medical School, Boston, USA; ^9^ present address: Wellcome Trust Sanger Institute, Wellcome Trust Genome Campus, Hinxton, Cambridgeshire CB10 1SA, UK

**Keywords:** ESRP1, RNA binding protein, proto-oncogene, human colorectal cancer

## Abstract

Epithelial splicing regulatory protein 1 (ESRP1) is an epithelial cell-specific RNA binding protein that controls several key cellular processes, like alternative splicing and translation. Previous studies have demonstrated a tumor suppressor role for this protein. Recently, however, a pro-metastatic function of ESRP1 has been reported. We thus aimed at clarifying the role of ESRP1 in Colorectal Cancer (CRC) by performing loss- and gain-of-function studies, and evaluating tumorigenesis and malignancy with *in vitro* and *in vivo* approaches. We found that ESRP1 plays a role in anchorage-independent growth of CRC cells. ESRP1-overexpressing cells grown in suspension showed enhanced fibroblast growth factor receptor (FGFR1/2) signalling, Akt activation, and Snail upregulation. Moreover, ESRP1 promoted the ability of CRC cells to generate macrometastases in mice livers. High ESRP1 expression may thus stimulate growth of cancer epithelial cells and promote colorectal cancer progression. Our findings provide mechanistic insights into a previously unreported, pro-oncogenic role for ESRP1 in CRC, and suggest that fine-tuning the level of this RNA-binding protein could be relevant in modulating tumor growth in a subset of CRC patients.

## INTRODUCTION

Colorectal cancer (CRC) is the third most common cancer worldwide [1]. Colorectal carcinogenesis is a complex process in which the activation of oncogenes and inactivation of tumor suppressor genes affect several critical cancer-related pathways [2, 3]. Recent advances in the field of CRC have highlighted several new key regulators of tumour initiation and progression, including short and long regulatory non-coding RNAs [4, 5]. Dysregulation in these molecules may alter many gene regulatory networks at the transcriptional, post-transcriptional or epigenetic level leading to cancer cell transformation.

RNA-binding proteins (RBPs) are also important for the genomic regulatory network within a cell and exert an array of functions, ranging from alternative splicing to mRNA translation and RNA degradation [6]. Due to their central role in RNA biogenesis, the expression of RBPs should be finely tuned in the cell [7]. Importantly, in several tissues, like the colon, RBPs are consistently and significantly highly expressed with respect to other classes of genes such as transcription factors, pointing out to the relevance of post-transcriptional regulation in maintaining homeostasis in these settings [7]. RBPs are emerging as key regulators of several processes in colon carcinogenesis. For instance, the RBP Quaking controls the differentiation of colon epithelium and acts as a suppressor of tumorigenesis [8]. Importantly, alterations in RBPs expression or mutation in the binding sites of target RNAs may lead to the formation of aberrant ribonucleoprotein complexes, thus changing their function and contributing to cancer initiation [9]. The RBP Musashi RNA-Binding Protein 2, for example, shows elevated expression in colorectal adenocarcinomas and promotes intestinal transformation [10]. The finding that RBPs can act both as oncogene or tumor suppressor, like Hu Antigen R, further complicates this issue [11].

Epithelial Splicing Regulatory Protein 1 (ESRP1) is an epithelial cell-specific RBP and splicing factor, which was first identified as a tumor suppressor in the colon adenocarcinoma cell line, LS180, due to its ability to bind to the 5′UTR of several cancer-related genes and regulate their translation [12]. The ESRP1 gene was also found to be the target of biallelic inactivating mutations in human colon cancers with microsatellite instability [13]. In agreement with the tumor suppressive role of ESRP1, several studies have shown that ESRP1 negatively regulates Epithelial-to-Mesenchymal Transition (EMT) in breast and pancreatic cancer, in oral squamous cell and non-small cell lung carcinomas [14,15,16,17]. Paradoxically, however, a pro-metastatic activity of ESRP1 has also been reported. ESRP1 expression in 4T1 breast cancer cells has been shown to enhance their metastatic potential and high ESRP1 expression is associated with poor survival of breast cancer patients [18]. Moreover, brain-metastatic progression of melanoma is positively correlated with the expression of CD44v6 isoform, a splicing target of ESRP1 [19]. More recently, Wang *et al*. described an association between copy number gains at three regions on chromosome 8, including 8q22 where ESRP1 is located and poor survival of gastric cancer patients [20].

In the present study, using both loss- and gain-of-function approaches, we demonstrate that ESRP1 plays a role in anchorage-independent growth of CRC cells. ESRP1-overexpressing CRC cells, when grown in suspension, show enhanced fibroblast growth factor receptor (FGFR1/2) signalling, Akt activation, and Snail upregulation. Furthermore, ESRP1 promoted the ability of CRC cells to generate macrometastases in mice livers. High ESRP1 expression may thus stimulate growth of cancer epithelial cells in the colon as well as at distant sites, and promote colorectal cancer progression.

## RESULTS

### ESRP1 is overexpressed in a subset of human CRC samples

**Figure 1:**
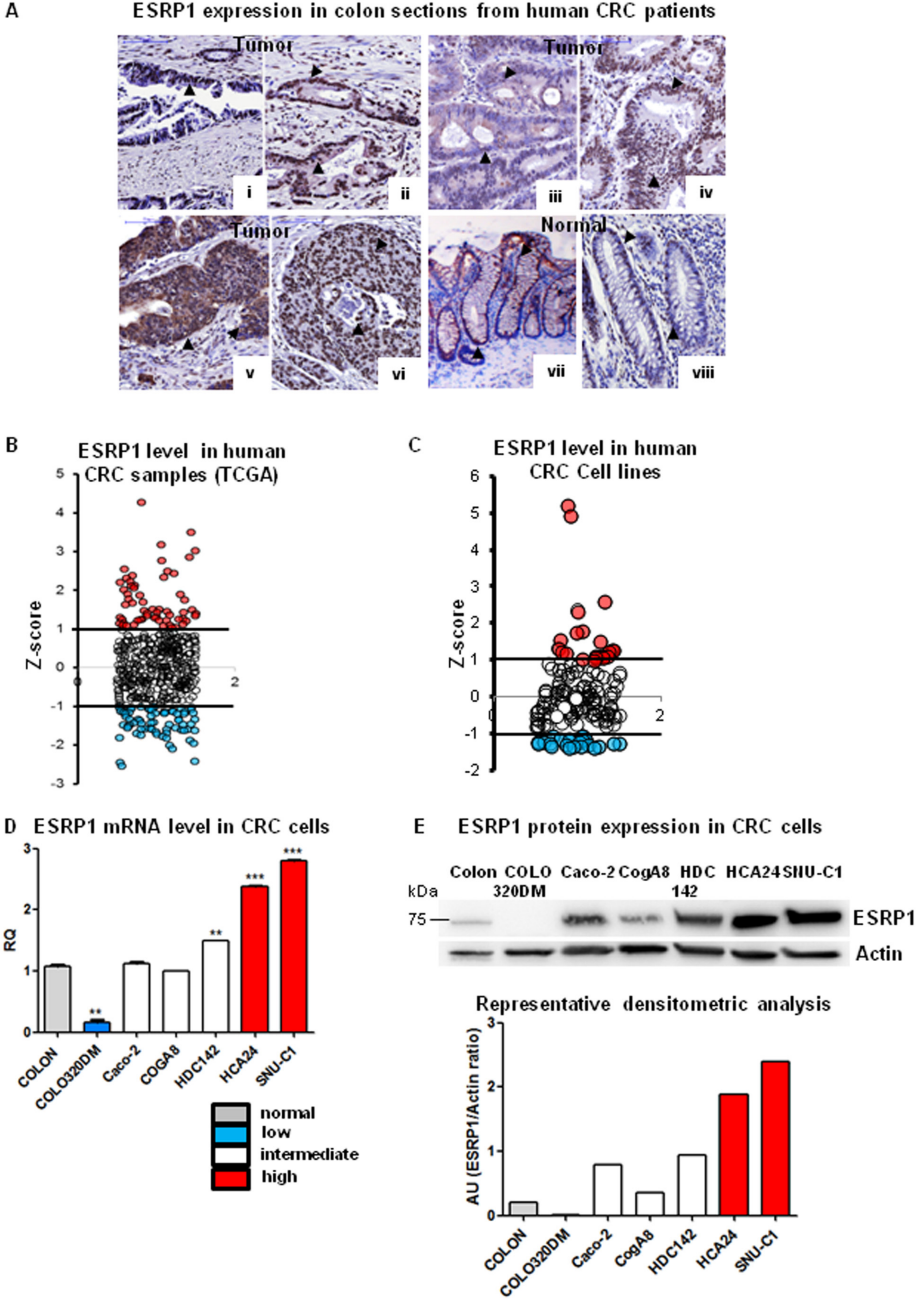
ESRP1 expression in CRC patients and cell lines **A**. Immunohistochemical analysis of ESRP1expression in human CRC sections and normal colonic mucosa: i-vi: tumor (grade I-III), vii-viii: normal colon (scale bar, 100μM). Arrowheads show ESRP1 positivity. **B**. Z-score of ESRP1 mRNA expression levels from TCGA RNA-seq data (450 tumor samples), showing tumors with high (red dots) and low (blue dots) ESRP1 expression. Z-score between 1 and -1 was considered normal. **C**. ESRP1 expression in 151 CRC cell lines were extracted from GEO and show cell lines with z-score >1 and <-1. **D**. qRT-PCR analysis of ESRP1 mRNA levels in selected cells lines versus normal colon (n=6; t-test of cell lines *versus* normal colon is shown). **E**. Representative western blot and densitometric analyses of ESRP1 in selected CRC cell lines versus normal colon (3 independent experiments).

To understand the role of ESRP1 in CRC, we examined ESRP1 expression in previously described Tissue Microarray (TMA) of primary human colon cancer by immunohistochemistry [21]. The intensity of ESRP1 expression in 75 out of 80 evaluable CRC sections stained was quite heterogeneous. Tumor sections showed moderate (nuclear or nuclear/cytoplasmic) (Figure 1A i-iv and supplementary Figure 1A) to high ESRP1 immunoreactivity (Figure 1A v-vi and supplementary Figure 1A) compared to normal human colon in which ESRP1 immunoreactivity was mainly found in the nuclei of cells of the mucosa (Figure 1A vii, viii and supplementary Figure 1A). Consistently, analysis of cancer genome atlas (TCGA) data from 450 human CRC samples revealed that ESRP1 expression was extremely heterogeneous, with RNA-Seq by Expectation Maximization (RSEM) values ranging from below 200 to over 8000. Based on ESRP1 mRNA expression (z-scores), 16% of the CRC samples had indeed elevated levels (z-score >1) and 13% of samples showed below-average ESRP1 expression (z-score <-1) (Figure 1B). Moreover, classification of ESRP1 expression *versus* molecular subtyping of CRC revealed that ESRP1 expression was elevated in some subtypes of tumors (Supplementary methods and Supplementary Figure 1B). In particular, C1 (Chromosomal Instability (CIN)_ImmuneDown_), C3 (*KRAS*-mutant) and C5 (CIN_WntUp_) molecular subtypes of CRC showed a mild but statistically significant increase in ESRP1 expression compared to the other subtypes (supplementary Figure 1B) [22].

As human CRC cell lines are relevant cancer models for studying gene function, we also interrogated our gene expression dataset, previously generated using a panel of 151 CRC cell lines, for ESRP1 expression [23]. In agreement with TCGA data, ESRP1 expression values ranged over more than one order of magnitude, with 15% of CRC cell lines expressing high levels (z-score >1) and 14% of cells expressing low levels (z-score <-1) (Figure 1C). We thus selected 6 CRC cell lines that expressed low (z-score < -1), intermediate (-1 ≥ z-score ≤ 1) or high (z-score >1) levels of ESRP1 for our *in vitro* studies, and ESRP1 expression was validated both at the RNA and protein levels (Figure 1D and E, respectively).

### ESRP1 promotes proliferation and tumorigenicity of CRC cells *in vitro*

**Figure 2:**
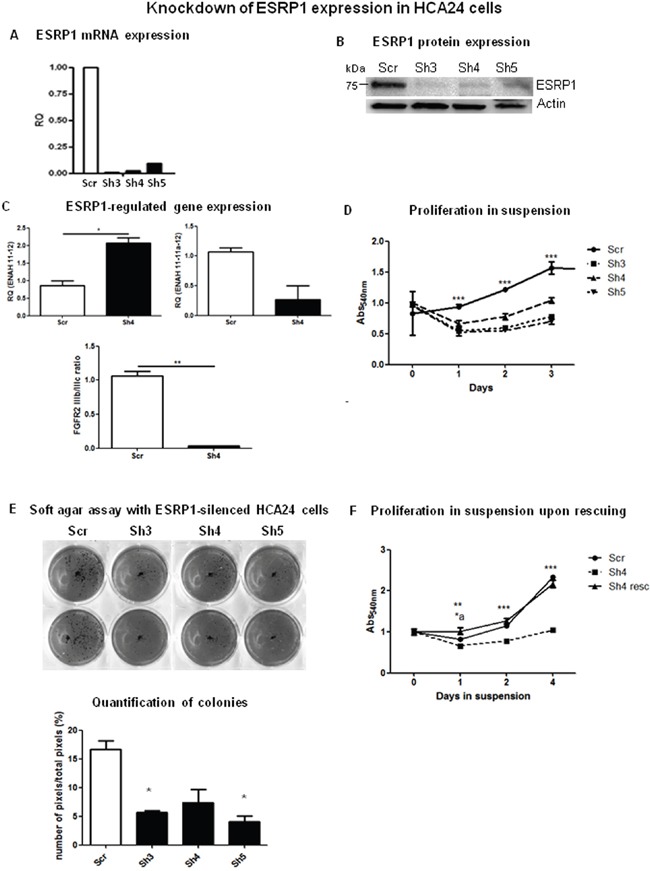
ESRP1-silencing reduces tumorigenicity of CRC cells **A**. Representative qRT-PCR and **B**. western blotting analyses of ESRP1 expression in ESRP1-silenced (Sh3, Sh4 and Sh5) and control (Scr) HCA24 cells. **C**. qRT-PCR analysis of ESRP1-regulated gene expression. **D**. MTT proliferation assays of ESRP1-silenced versus Scr control HCA24 cells grown in suspension (n=8, 2 independent experiments). **E**. Soft agar assay with ESRP1-silenced and control HCA24 cells (n=3, 2 independent experiments) and Image J software quantification of pixels/well (n=6). **F**. MTT proliferation assays of ESRP1-silenced (Sh4) versus Scr control and Sh4 rescued HCA24 cells grown in suspension (n=6, 2 independent experiments, *a is t-test comparing Scr *vs* Sh4 resc, **, *** is t-test comparing Sh4 *vs* Scr and Sh4 *vs* Sh4 resc).

ESRP1 has been described as a tumor suppressor in several types of cancers [14,15]. We thus knocked down ESRP1 in HCA24 cells (ESRP1^high^) to investigate the effect of ESRP1 silencing on CRC progression. HCA24 cells were infected with lentivirus prepared with three different shRNA sequences (Sh3, Sh4 and Sh5) giving efficient knockdown of ESRP1 expression as confirmed by analysis of mRNA (Figure 2A) and protein (Figure 2B) levels. The specificity of the shRNAs was verified by analyzing the expression of the alternatively spliced isoforms of ESRP1 target genes, ENAH [Enabled Homolog (Drosophila)] and FGFR2, and as expected, there was an increase in the mesenchymal isoforms (ENAH 11-12 and FGFR2 IIIc) upon ESRP1 silencing in HCA24 cells (Figure 2C and supplementary Figure 1C). Surprisingly, in HCA24 cells, constitutive knockdown of ESRP1 abrogated the growth in suspension (Figure 2D) and reduced the anchorage-independent growth *versus* Scr controls (Figure 2E). We performed a rescue experiment by substituting 3 bases in three different codons of the Sh4 binding site present in the ESRP1 overexpression construct. Transfection of the mutant construct in ESRP1-silenced HCA24 (Sh4) cells rescued the anchorage-independent growth ability as well as ESRP1-regulated gene expression of these cells to levels comparable to Scr controls (Figure 2F and supplementary Figure 2A, respectively). ESRP1 silencing in another transformed CRC cell line, HDC142 (ESRP1^intermediate^) also abolished their colony-forming capacity in soft agar (supplementary Figure 2B). These data indicate that constitutive silencing of ESRP1 expression reduced anchorage-independent CRC cell growth.

**Figure 3:**
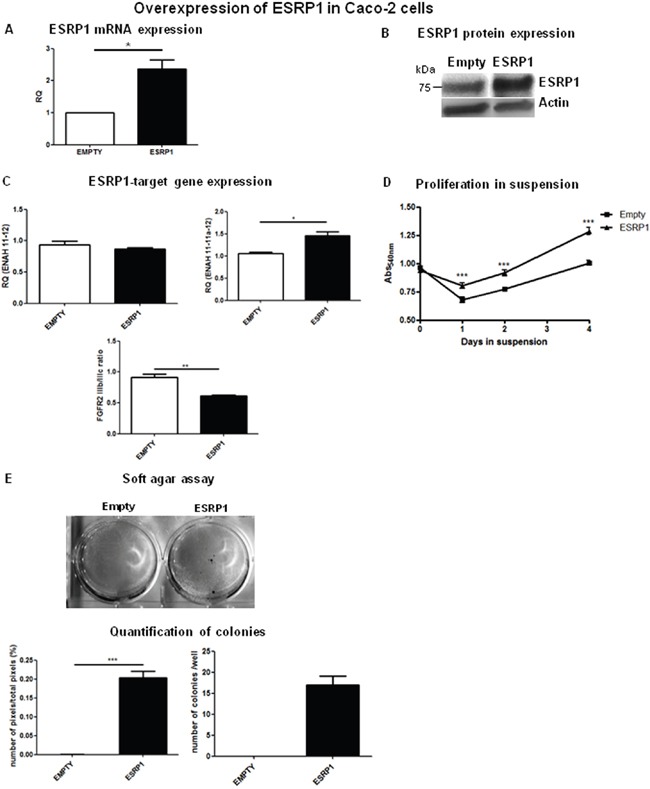
ESRP1 overexpression promotes proliferation and transformation of Caco-2 cells **A**. qRT-PCR and **B**. western blotting analyses of ESRP1 expression in Caco-2 cells (Empty controls versus ESRP1-overexpressing (ESRP1)). **C**. qRT-PCR analysis of ESRP1-regulated gene expression. **D**. MTT proliferation assays of ESRP1-overexpressing Caco-2 cells versus Empty controls grown in suspension (n=8, 2 independent experiments). **E**. Soft agar assay performed with ESRP1-overexpressing and control Caco-2 cells (6 independent experiments), Image J software quantification of pixels/well and colony counts.

**Figure 4:**
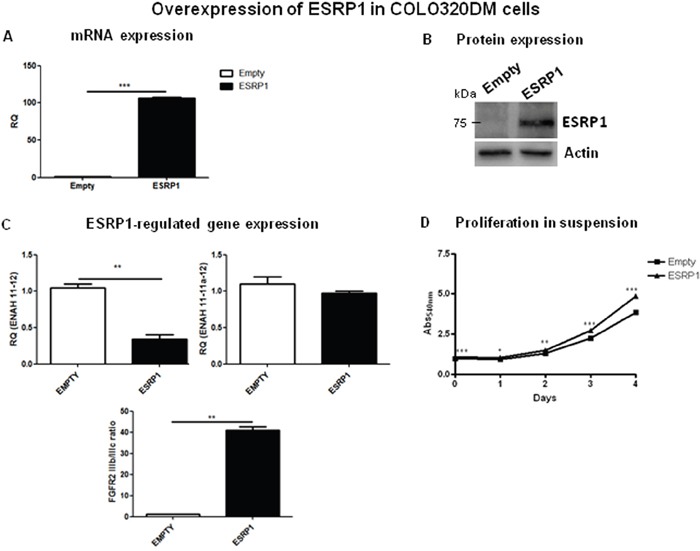
Overexpression of ESRP1 in COLO320DM cells **A**. qRT-PCR and **B**. western blotting analyses of ESRP1 expression in ESRP1-overexpressing and control COLO320DM cells (n=2, 2 independent experiments). **C**. qRT-PCR analysis of ESRP1-regulated gene expression. **D**. MTT proliferation assays of ESRP1-overexpressing COLO320DM cells versus Empty controls grown in suspension (n=8, 2 independent experiments).

To investigate a potential oncogenic role for ESRP1 in CRC, we chose Caco-2 cells, a normal-like colon cell line (ESRP1^intermediate^), to perform both loss- and gain-of-function experiments. Upon ESRP1-silencing, proliferation in suspension (supplementary Figure 3) or anchorage-independent growth (not shown) of Caco-2cells, which usually do not grow in anchorage-independency, did not change *versus* Scr controls. We next stably overexpressed ESRP1 in the non-transformed Caco-2 cells, and overexpression was confirmed both at mRNA (Figure 3A) and protein (Figure 3B) levels. Analysis of ESRP1-regulated genes, ENAH and FGFR2, showed that there was a statistically significant increase in the expression of the epithelial isoform of the former (ENAH 11-11a-12), but a slight decrease in the FGFR2 IIIb/IIIc (epithelial/mesenchymal) ratio (Figure 3C). Remarkably, elevated ESRP1 expression promoted the proliferation of Caco-2 cells in suspension (Figure 3D) and colony formation in soft agar assay after 60 days of culture compared to the Empty controls, thus indicating a role for ESRP1 in the anchorage-independent growth of Caco-2 cells (Figure 3E). Moreover, we restored ESRP1 expression (Figure 4A and 4B) in an ESRP1-null COLO320DM cells (ESRP1^low^) presenting poorly-differentiated features and growth in semi-suspension. Analysis of ESRP1-regulated genes showed that there was a statistically significant decrease in the expression of the epithelial isoform of ENAH, and a significant increase in the FGFR2 IIIb/ IIIc (epithelial/mesenchymal) ratio (Figure 4C). Again, ESRP1-expressing COLO320DM cells showed a slight but statistically significant increase in proliferation in suspension cultures compared to Empty controls (Figure 4D) confirming the data obtained in ESRP1-overexpressing Caco-2 cells. Overall, *in vitro* analysis in 4 different colon cancer cell lines indicated a pro-oncogenic role of ESRP1 in CRC, in particular in sustaining anchorage-independent growth and transformation.

### ESRP1 enhances primary tumor growth *in vivo*

**Figure 5:**
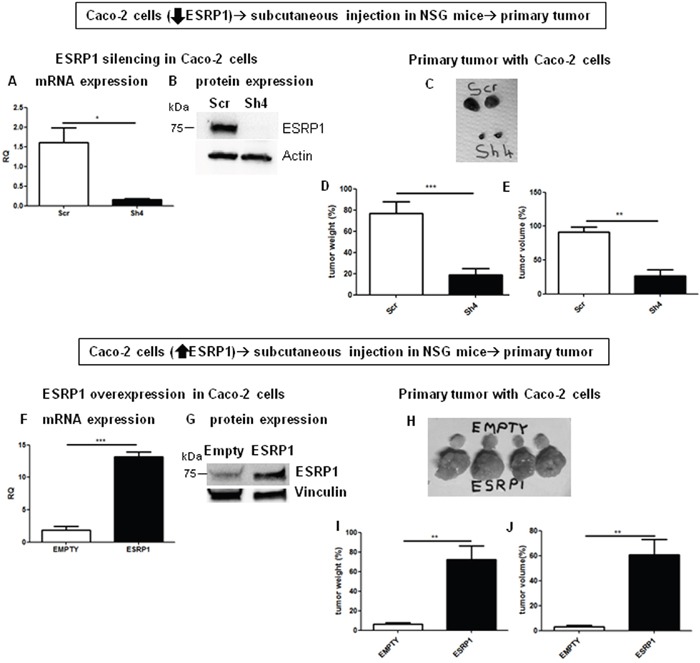
ESRP1 overexpression promotes tumor growth in NSG mice *in vivo* **A**. qRT-PCR and **B**. western blotting analyses of ESRP1 expression in ESRP1-silenced Caco-2 cells. **C**. Representative tumors are shown. Tumor **D**. weight and **E**. volume are shown in the graphs (n=8, 2 independent experiments). **F**. qRT-PCR and **G**. western blotting analyses of ESRP1 expression in ESRP1-overexpressing and control Caco-2 cells. **H**. Representative tumors are shown. **I**. Tumor weight and **J**. volume are shown in the graphs (n=8, 2 independent experiments).

We further confirmed the *in vitro* results by performing xenograft assays with ESRP1-silenced and -overexpressing Caco-2 cells. Caco-2 cells were injected subcutaneously in NOD/SCID/gamma-null (NSG) mice which were monitored weekly. Visible tumors formed 45 days after cell injection and grew very fast thereafter, and all tumors were dissected 60 days after cell injection. The results showed that while ESRP1-silenced tumors were significantly smaller compared to Scr control tumors (Figures 5A to 5E), ESRP1-overexpressing Caco-2 cells generated significantly larger tumors compared to Empty controls (Figures 5F to 5J). Altogether, these findings strongly support an important role for ESRP1 in promoting tumor growth.

### ESRP1–driven transformation is PI3K/Akt-dependent

**Figure 6:**
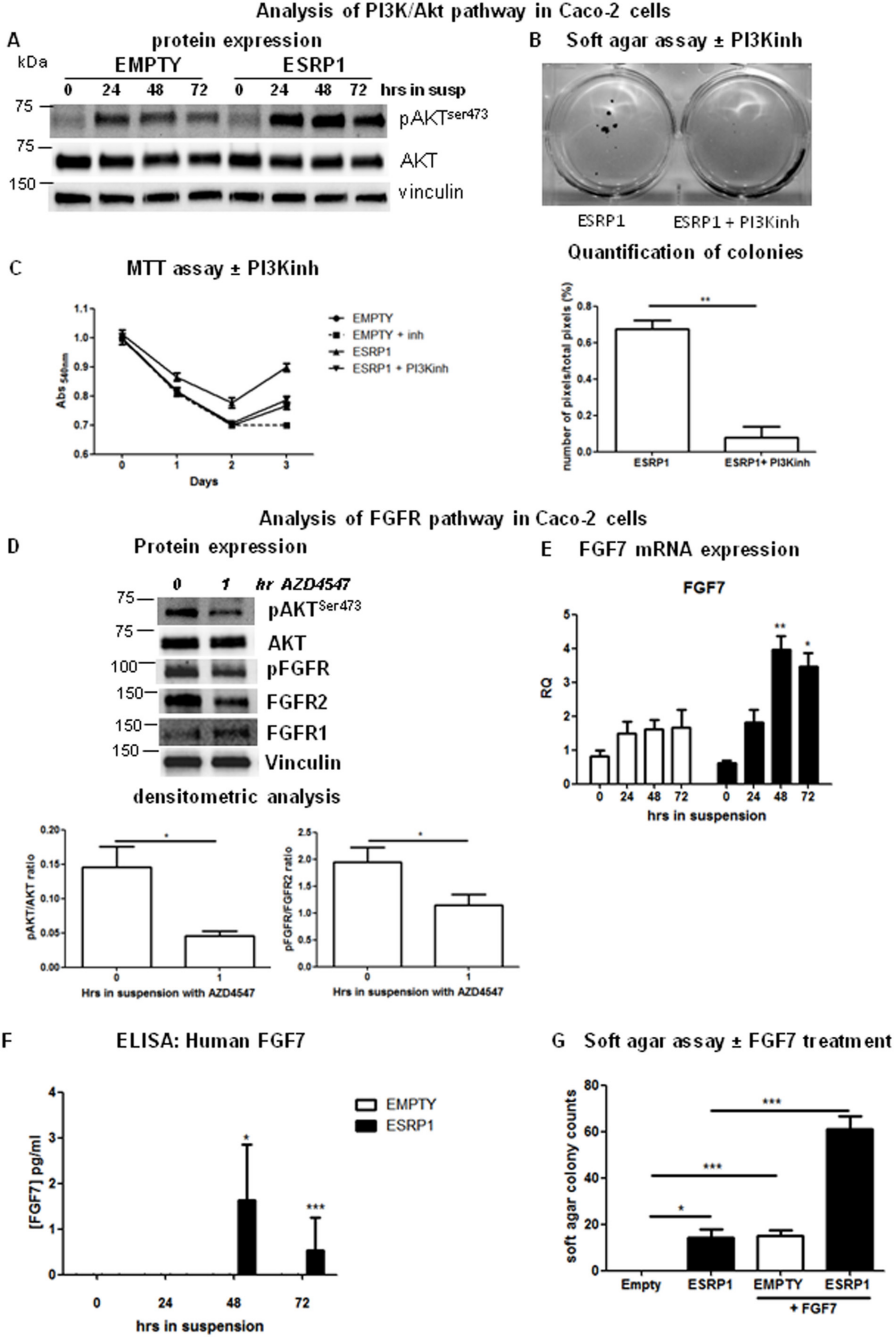
Pathways involved in ESRP1-driven CRC cell transformation **A**. Western blot analysis of phospho-AKT (pAKT^ser473^) expression in ESRP1-overexpressing Caco-2 cells versus Empty controls following growth in suspension for 72 h. Images are representative of 3 independent experiments. **B**. Soft agar assay and Image J software quantification of pixels/well (n=6) of ESRP1-overexpressing Caco-2 cells untreated or treated with PI3K inhibitor ZSTK474 (inh) (n=3, 2 independent experiments). **C**. MTT assay proliferation of ESRP1-overexpressing Caco-2 cells following PI3K inhibition versus Empty controls. **D**. Western blot and densitometric analyses of pAKT^ser473^, total AKT, phospho-FGFR^Tyr6537654^, total FGFR1 and FGFR2 expression in ESRP1-overexpressing Caco-2 cells grown in suspension untreated and treated for 1h with FGFR inhibitor (3 independent experiments). **E**. qRT-PCR analysis of FGF7 mRNA expression in ESRP1-overexpressing and control Caco-2 cells grown in suspension for 72 h (n=6, 3 independent experiments). **F**. ELISA for human FGF7 was performed in serum-free culture supernatant of ESRP1-overexpressing and control Caco-2 cells grown in suspension for 72 h (3 independent experiments). **G**. Soft agar assay colony counts of Empty and ESRP1-overexpressing Caco-2 cells, treated with FGF7 versus non-treated controls (n=3, 2 independent experiments).

As Akt is a potent survival factor in colorectal carcinogenesis, we next addressed whether the PI3K/Akt signaling pathway is involved in the phenotype observed in ESRP1-overexpressing Caco-2 cells [24]. Interestingly, when ESRP1-overexpressing Caco-2 cells were grown in suspension for 24 h, cell aggregates formed, and Akt was significantly more phosphorylated at Ser^473^, reflecting a higher Akt activity, compared to basal conditions and Empty controls. This increase remained constant in the suspension cultures up to 72 h (the last analyzed time point, Figure 6A). Treatment of ESRP1-overexpressing Caco-2 cells with ZSTK474, a selective PI3K inhibitor, significantly reduced colony formation in soft agar (Figure 6B) and rescued the enhanced growth in suspension to levels comparable to Empty controls (Figure 6C), showing that the PI3K/Akt pathway was implicated in ESRP1-driven Caco-2 cell survival and transformation [25].

### The oncogenic effects of ESRP1 on CRC cells are dependent on FGFR activation

As the receptor tyrosine kinase (RTK) Fibroblast Growth Factor Receptor (FGFR) 2 is a known splicing target of ESRP1 and is highly expressed in some CRC cell lines, we investigated whether the FGFR pathway was involved in the activation of Akt in the ESRP1-overexpressing cells [23,26]. In Caco-2 cells grown in suspension, we did not find any significant difference in the FGFR2IIIb/IIIc ratio between ESRP1-overexpressing cells and Empty controls up to 72 h (supplementary Figure 4A). However, further analysis of the FGFR activation (phosphorylation at Tyr^653^ and Tyr^654^) in these cells showed that FGFR was more phosphorylated at 24 h in suspension in ESRP1-overexpressing Caco-2 cells compared to Empty controls (supplementary Figure 4B). Treatment of ESRP1-overexpressing Caco-2 cells (grown for 24 h in suspension) with AZD-4547, a FGFR1/2 inhibitor, resulted in significantly reduced FGFR phosphorylation as well as in reduced AKT^Ser473^ phosphorylation at 1 hr post-treatment showing that the FGFR pathway was activated in these cells (Figure 6D). Moreover, the FGFR substrate 2 and adaptor protein, FRS2, also showed enhanced expression as well as phosphorylation in ESRP1-overexpressing Caco-2 cells with respect to Empty controls following growth in suspension for 24 h (supplementary Figure 4B). Knockdown of FGFR2 expression in ESRP1-overexpressing Caco-2 cells reduced the size of the soft agar colonies showing that FGFR2 participated in anchorage-independent tumor growth (supplementary Figure 4C).

**Figure 7:**
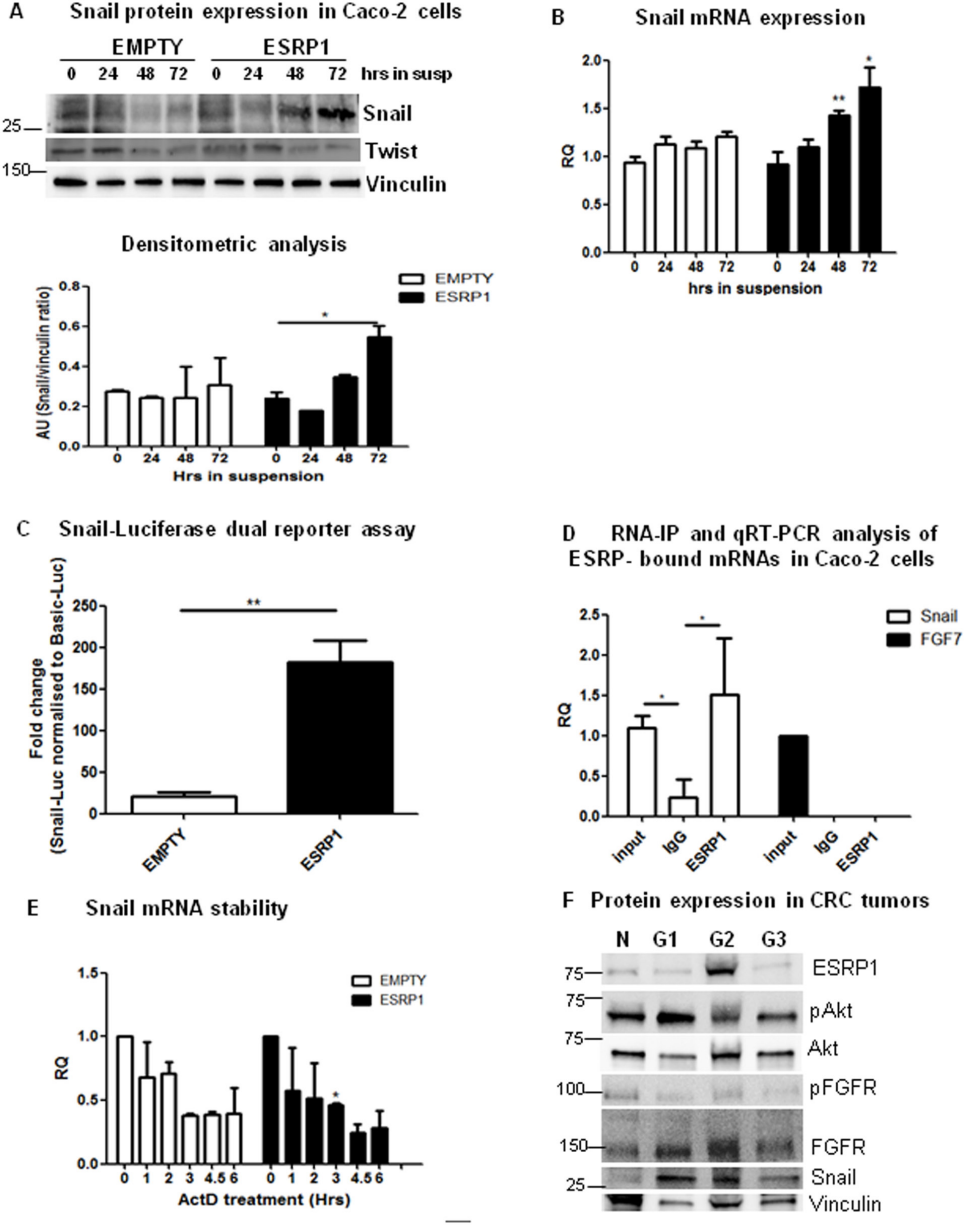
ESRP1 overexpression induces Snail expression and enhances metastasis formation **A**. Western blot, densitometric and **B**. qRT-PCR analyses of Snail expression in ESRP1-overexpressing and control Caco-2 cells grown in suspension for 72 h (3 independent experiments). **C**. Snail-Luciferase dual reporter assay performed in Empty and ESRP1-overexpressing Caco-2 cells grown in serum-free media and in suspension for 72 h. Fold change with respect to basal-LUC is shown (n=4, 2 independent experiments). **D**. RNA-IP performed on ESRP1-overexpressing Caco-2 cells and qRT-PCR analysis of ESRP1-bound transcripts (n=4, 2 independent experiments). **E**. qRT-PCR analysis of Snail mRNA stability following actinomycin D treatment (2 independent experiments). **F**. Western blot analysis of representative CRC tumor samples, classified according to grading (N: Normal, G1: Grade I, G2: Grade II, G3: Grade III).

Ligands causing FGFR activation are usually produced by stromal cells, but tumor cells can also establish an autocrine loop to aberrantly activate the FGFR pathway. We thus analyzed the expression of FGFR ligands specific for FGFR2IIIb (FGF7 and FGF10) and FGFR2IIIc (FGF2 and FGF18) isoforms. While FGF10 and FGF18 were not expressed by Caco-2 cells, FGF7 mRNA expression was significantly increased in ESRP1-overexpressing cells in suspension compared to Empty controls (Figure 6E). FGF2 (which activates both FGFR1 and FGFR2IIIc) expression was higher in ESRP1-overexpressing Caco-2 cells under basal conditions as well as in suspension cultures (Supplementary Figure 4) compared to Empty controls. Further analysis at protein level showed that secreted FGF7, which binds specifically the ESRP1-promoted FGFR2IIIb isoform, was detectable in the culture supernatant of ESRP1-overexpressing Caco-2 cells following growth in suspension for 48 h (Figure 6F), suggesting an autocrine activation of FGFR2IIIb in these cells. In the ESRP1-overexpressing cells, FGF7 mRNA (half-life: 140 mins) showed enhanced stability compared to other mRNAs analyzed (Supplementary Figure 5) [27]. However, ESRP1did not bind FGF7 mRNA as revealed by RNA-IP experiment, suggesting an indirect control of its stability (Figure 7D and Supplementary Figure 5B). Importantly, treatment of Empty control Caco-2 cells with FGF7 resulted in colony formation in soft agar assay compared to non-treated cells (Figure 6G). FGF7 treatment also significantly enhanced the growth of ESRP1-overexpressing Caco-2 cells in soft agar as evidenced by the increase in number of colonies with respect to non-treated cells. These data show that ESRP1 overexpression caused increased FGFR ligand production and receptor activation when Caco-2 cells were grown in suspension.

### ESRP1 overexpression enhances formation of macrometastases from CRC cells

The PI3K/Akt pathway, through the phosphorylation of numerous substrates, is associated with processes underlying EMT and metastasis of cancer cells [28]. In particular, phosphorylation of one of these substrates, GSK3β, by Akt at Ser^9^ inhibits its activity and leads to an enhanced stabilization of the zinc-finger protein, Snail [29]. We thus analyzed the expression of Snail in our system. We found that Snail was expressed in ESRP1-overexpressing Caco-2 cells grown in suspension in serum-free media for 48 h compared to Empty controls (Figure 7A). We also observed a significant increase in Snail mRNA level in ESRP1-overexpressing cells in suspension cultures as from 48 h (Figure 7B). Caco-2 cells transfected with a Snail-luciferase construct and subsequently grown for 72 h in suspension were analysed in a dual reporter assay. The results show that Snail protein activity was significantly enhanced in ESRP1-overexpressing cells compared to Emtpy controls and with respect to adherent cells (Figure 7C and supplementary Figure 8, respectively). Interestingly, RNA-IP analysis revealed that ESRP1 was able to bind Snail mRNA (Figure 7D) without affecting its stability (Figure 7E). These data suggest that ESRP1 could regulate Snail transcription and/or translation, hence potentiating the effect of Akt activation on Snail expression in the ESRP1-overexpressing Caco-2 cells. The expression of Twist, another member of the helix-loop-helix and zinc-finger protein families, did not differ between the two conditions both at protein (Figure 7A) and RNA levels (not shown). Analysis of human CRC samples, classified according to grading, further showed that Snail protein was expressed in tumors versus normal samples (Figure 7F). Importantly, the results show that ESRP1 and Snail may be co-expressed in human CRC, as seen in the G2 sample analysed, hence supporting our data on ESRP1-overexpression in Caco-2 cells.

**Figure 8:**
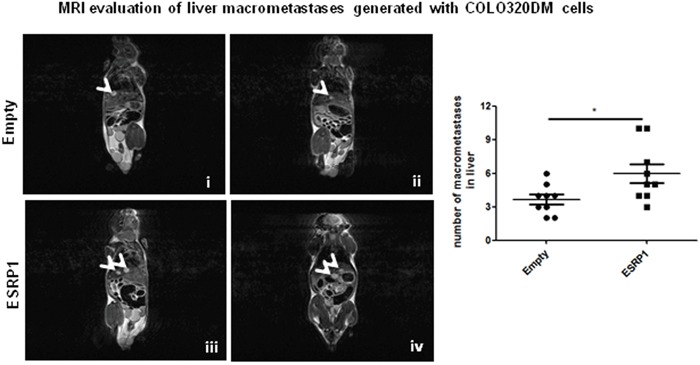
Effect of ESRP1 overexpression on metastatic potential of CRC cells *in vivo* Representative MRI T_2w_ images of NSG mice injected with COLO320DM cells showing liver macrometastases (arrowheads). Bar graphs of liver macrometastases, n=9 for each group.

We further investigated the effect of ESRP1 overexpression on the metastatic process *in vivo*. As ESRP1-overexpressing Caco-2 cells did not form significant metastasis *in vivo* (Supplementary Figure 6), we employed another highly metastatic CRC cell line, COLO320DM, for experimental metastasis. Three weeks after intravenous cell injection, COLO320DM cells formed macrometastases in the liver of NSG mice as revealed by MRI analysis. ESRP1-overexpressing COLO320DM cells resulted in a significantly larger number of macrometastases compared to Empty controls (Figure 8).

**Figure 9:**
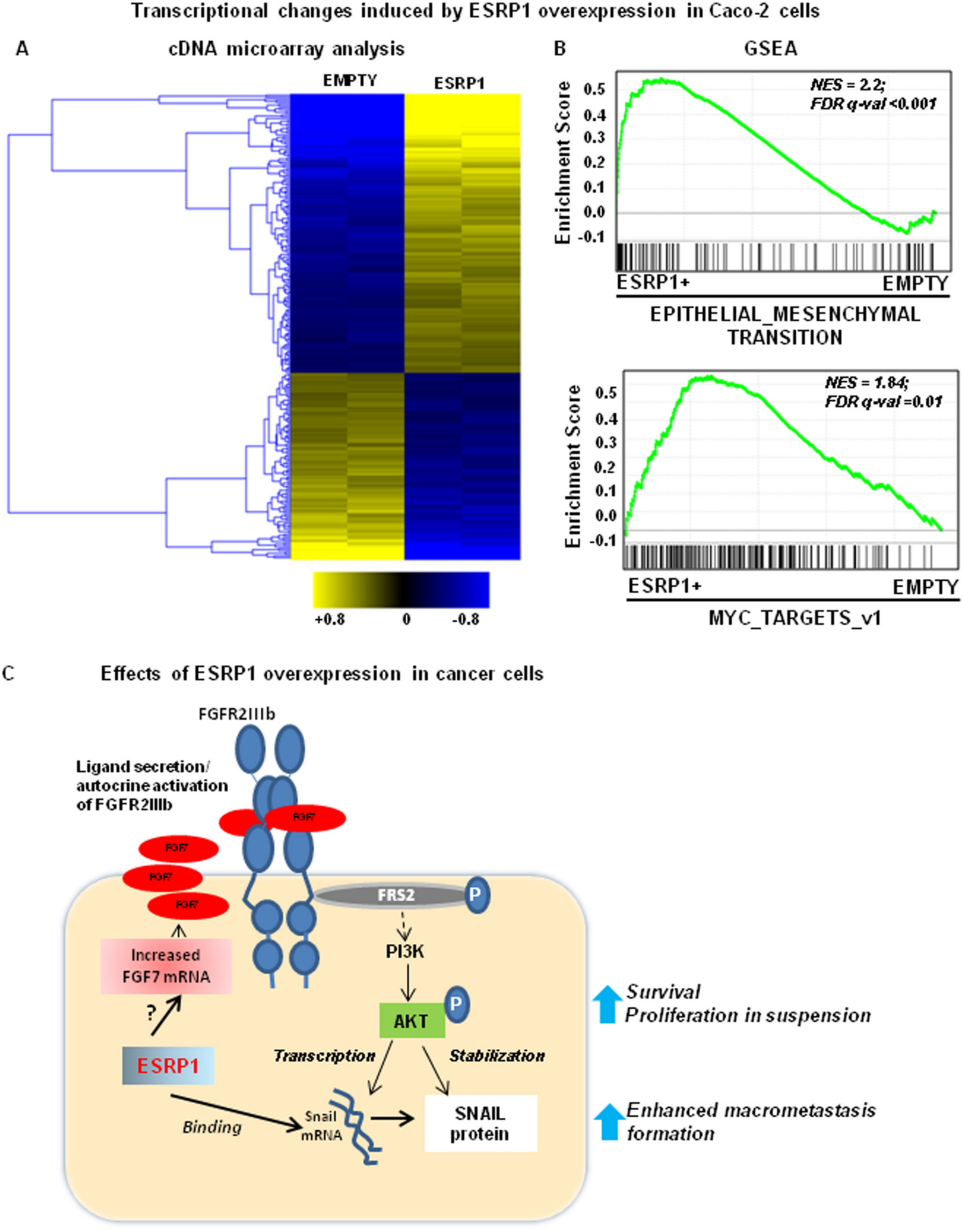
Effects of ESRP1 overexpression on cancer cells **A**. Gene expression profiling of ESRP1-overexpressing and control adherent Caco-2 cells. The heatmap reports the expression (log_2_ ratio against average). **B**. GSEA of the transcription profiles shows enrichment in gene signatures in ESRP1-overexpressing Caco-2 cells *versus* Empty controls. **C**. The cartoon depicts the potential effects of ESRP1 overexpression in CRC cells.

To gain further mechanistic insights into the molecular changes caused by ESRP1 modulation, we performed gene expression profiling of Caco-2 cells grown as monolayer (ESRP1-overexpression and ESRP1-silencing, Figure 9A and supplementary Figure 7A, respectively). Selected differentially expressed genes (Supplementary Table 3) were validated at mRNA level (Supplementary Figure 7C). Gene Set Enrichment Analysis (GSEA) of these changes revealed that EMT and Myc targets were among the most significantly enriched terms in ESRP1-overexpressing cells (Figure 9A) compared to control cells. On the other hand, we found that the expression of EMT-related genes was reduced in ESRP1-silenced Caco-2 cells with respect to Scr controls (supplementary Figure 7B).

To deeply investigate the modulation of EMT signature genes after ESPR1 overexpression, we extracted expression level of all the transcripts present in EMT gene set from our cDNA microarray data performed on Caco-2 cells under adhesion (basal conditions). According to GSEA results, the majority of these genes (63%) were upregulated in ESRP1-overexpressing Caco-2 cells (Supplementary Table 4). Moreover, 6 of these were also selected in the ESRP1 signature and, in particular, PMP22 and VCAN transcripts were ranked among the top genes induced by ESRP1 overexpression (supplementary Figure 7C).

We also examined Akt pathway in the RNA profile by performing *in silico* functional analysis (see supplementary methods). GSEA revealed that Akt pathway gene set was not significantly enriched in ESRP1-overexpressing Caco-2 cells (FDR q-val = 0.87), while IPA upstream analysis showed a weak but significant activation of AKT signaling pathway (p = 1.88E-02) inferred by the positive modulation of Akt target genes in ESRP1-overexpressing Caco-2 cells. Overall, these results based on mRNA profile suggest that Akt pathway is not markedly upregulated in adherent cells after ESRP1 overexpression. This is in line with the results obtained by Western blot in which we were able to highlight AKT phosphorylation only when cells were kept in suspension.

ESRP1-overexpressing cells showed enhanced survival in suspension cultures through Akt activation, Snail expression as well as other EMT-linked alterations, thus promoting CRC cell growth in anchorage-independency *in vitro* and macrometastasis formation *in vivo*.

## DISCUSSION

RBPs are part of the largest group of proteins that orchestrates the passage of RNA through the RNA regulon and may coordinate the expression of diverse genes responsible for cell growth and proliferation. Thus, aberrant expression of RBPs might alter cell physiology and lead to cancer development [9]. As an RBP, ESRP1 is involved in several cellular processes like alternative splicing, regulation of translation and mRNA stability [30,31]. ESRP1 is a documented tumor suppressor but our analysis on CRC cell lines and TCGA expression datasets also revealed cases in which this RBP was overexpressed [32]. In an attempt to clarify the role of ESRP1 in CRC progression, we modulated the level of this protein in four cell lines and found a previously unreported, pro-tumorigenic function of ESRP1 in CRC. Our data provide the first evidence that ESRP1 is required for CRC cell anchorage-independent growth *in vitro* and tumor growth *in vivo*. Regarding the underlying mechanism, we show that, when Caco-2 cells are grown in suspension, ESRP1 indirectly causes an increase in the secretion of FGF7 and FGF2, leading to FGFR activation and sustained Akt phosphorylation. FGFR or PI3K/Akt inhibition reverted the pro-oncogenic phenotype observed in Caco-2 cells upon ESRP1 overexpression.

In ESRP1-overexpressing Caco-2 cells, FGF7 and FGF2 production participated to an autocrine activation of FGFR (FGFR1 and FGFR2), and addition of exogenous FGF7 to Caco-2 cells in anchorage-independent growth assay induced colony formation in Empty controls and increased the number of colonies in ESRP1-overexpressing cells. Binding of RNA targets by ESRP1 usually results in their translational suppression without affecting overall mRNA stability [12,31]. Our native RNA-IP of ESRP1-bound transcripts in Caco-2 cells showed that FGF7 mRNA was not bound by ESRP1. Thus, the increased stability of FGF7 and FGF2 mRNA in these cells was probably enhanced by other stimuli or mechanism warranting further studies, for example through other regulatory RNAs like miRNAs [33]. These ligands, usually secreted by stromal cells surrounding the tumor, can also be produced by advanced cancer cells [34]. Autocrine signalling by many growth factor-receptor combinations is a major stimulus of cell transformation and, several studies have shown that the creation of a FGF7 autocrine loop provides conditions in which subsequent changes can occur culminating in malignancy [35]. Moreover, aberrant FGF signalling and FGFR activation induce proliferation and survival of tumor cells [36,37]. Phosphorylated FGFR can lead to the activation of multiple signal transduction pathways, involving key adaptor proteins, among which FRS2. One of the complexes recruited to phosphorylated FRS2 includes growth factor receptor-bound 2-associated binding protein 1 (GAB1) and PI3K which activates Akt-dependent survival pathway. Our results showed higher expression of FRS2 as well as of phosphorylated FRS2 in ESRP1-overexpressing Caco-2 cells in suspension cultures *versus* Empty controls.

In CRC, Akt activation is associated with increased proliferation and loss of epithelial differentiation [38]. Activated Akt phosphorylates several substrates, like GSK3β, that are involved in essential cell processes like survival and protein translation [24]. Interestingly, Akt, through inhibition of GSK3β, can stabilize Snail protein which, apart from its well-documented role as inducer of EMT, also participates in cell survival, immune regulation and stem cell biology [39]. Akt can act through phosphorylation of GSK3β or IKKα/IKK β/NF-κB pathway to promote Snail stability and transcription, respectively [40,41]. It has been shown by Wang *et al*. that suppression of Akt can result in a distinct reduction of Snail expression at mRNA level, hence supporting our data [42]. In accordance, we detected an increased level of Snail mRNA in ESRP1-overexpressing cells grown in suspension. Moreover, our RNA-IP data show that ESRP1 can bind Snail mRNA, but has no effect on mRNA stability, suggesting that ESRP1 could be regulating the translation of Snail. Of note is the fact that during carcinogenesis, Snail mRNA can be also stabilized by other RBPs like Human antigen R (HuR) [43]. In immortalized human mammary epithelial cells, Reinke *et al*. have shown that Snail can regulate the transcription of ESRP1, thus facilitating EMT and tumor progression [44]. We obtained similar results in adherent cells using Snail-luciferase reporter assay. Our data also show that overexpression of ESRP1 led to detectable Snail protein expression when cells were grown in suspension without serum *versus* Empty controls. The combined effects of Akt and FGFR activation upon ESRP1 overexpression may account for the increased Snail gene expression in the ESRP1-overexpressing Caco-2 cells, and may be responsible for the inactivation of the inhibitory loop between ESRP1 and Snail. Furthermore, our data show ESRP1 can co-express with Snail in some human CRC samples. Snail expression has been found upregulated in 60–70% of CRC, and is associated not only with poor prognosis, but also with shortened relapse-free survival [45]. We further analysed whether pAKT, pFGFR and Snail were involved in the phenotype observed in ESRP1-silenced HCA24 cells grown in suspension but found that there were no differences between ESRP1-silenced cells compared to Scr control (not shown). This indicates that different mechanisms were responsible for the behaviour of ESRP1-silenced and-overexpressing cells in anchorage-independency.

We also observed a significantly higher number of macrometastases in the liver of NSG mice with ESRP1-overexpressing COLO320DM cells compared to Empty controls. This may be due to the ability of ESRP1 to promote tumor growth *in vivo*. ESRP1-induced changes, such as Snail expression, did not affect liver colonisation of CRC cells as reflected by similar number of micrometastases generated by ESRP1-overexpressing and Empty control COLO320DM cells, hence supporting the data showing that Snail is dispensable for metastasis, as occurs in pancreatic cancer [46]. How ESRP1 can promote metastatic cell growth *in vivo* warrants further investigation.

The work of Leontieva *et al*. showed that ectopic expression of ESRP1 (RBM35A) in ESRP1-null LS180 colon carcinoma cells inhibited anchorage-independent growth *in vitro* and suppressed tumorigenic potential *in vivo* [12]. This apparent contrast could be due to the fact that the authors used a Tet-off regulated system to obtain physiological level of ESRP1 in LS180 cells compared to our constitutive overexpression of ESRP1. During the revision of our work, a paper was published in which the expression of ESRP1 and ESRP2 as well as ESRPs-mediated alternative splicing patterns in CRC were analysed [47]. The authors showed that CRC cells with reduced expression of ESRPs showed splicing patterns associated with EMT. Especially, reduced ESRP1 expression switches FGFR2 expression to more mesenchymal splice variants with a strong potential of disease progression, and showed a correlation between ESRPs expression and a favorable outcome in CRC. These are not surprising considering the evidences showing that a number of genes, often coding for multifunctional proteins like ESRP1, show oncogenic activities both when over- or under-expressed, highlighting a need for fine tuning of their expression levels in normal cells [48].

In conclusion, our data show, for the first time, that aberrantly high ESRP1 expression can drive tumor progression in CRC. RBP activity, in contrast with transcription factors, is restricted to the repertoire of transcripts expressed in a given cell type [10]. Thus, RBPs may serve as a hub of signal integration by acting on mRNAs available for binding in a given cell type hence performing cell-type-specific function. The autocrine activation of FGFR2, enhanced Akt activation, increased Snail expression in CRC cells constitutively overexpressing ESRP1, as depicted in Figure 9C, may instigate cell protection mechanisms causing cell survival in suspension cultures. Maintaining a finely tuned expression of ESRP1 is important in epithelial cells as elevated ESRP1 levels may post-transcriptionally alter expression of genes involved in pathways that synergize to drive cancer, as happens in a subset of CRC.

## MATERIALS AND METHODS

### CRC cell lines

The CRC cell lines used in this study were tested and authenticated, and characterized by genetic and transcriptional profiling as we previously reported in 2015 [23]. Culture media used are described in supplementary methods. Cells were treated with 10^-9^ M FGF7 (Peprotech) [49], 5μM of the FGFR inhibitor, AZD4547 (Selleck Chemicals) or 1μM of the PI3K inhibitor, ZST K474 (kind gift from E. Ciraolo).

### Stable ESRP1 knockdown and overexpression in CRC cell lines

Five shRNA (TRCN0000240872- TRCN0000240875 and TRCN0000240878, Openbiosystems) were analyzed for efficient knockdown of Esrp1 in HCA24 cells using Lipofectamine 2000 (Invitrogen) and according to manufacturer’s instructions. Lentivirus was produced in 293FT cells as previously described [31]. For overexpression of ESRP1 and lentivirus production, human ESRP1 ORF (HORFEOME v8.1, OpenBiosystems) was cloned into pLX304 by gateway recombination. After infection, cells were selected using puromycin (pLKO.1) or blasticidin (pLX304) prior to performing experiments.

### Luciferase reporter gene assays

Basic-LUC or Snail-LUC (Snail_pGL2 was from Paul Wade (Addgene plasmid # 31694)) and Renilla plasmids were transfected into Caco-2 cells using Lipofectamine 2000 (Invitrogen) according to the manufacturer's guidelines [50]. After transfection, cells were grown in suspension in serum-free media for 72h. Luciferase reporter gene assays were conducted using the Luciferase Assay System (Promega) and its corresponding protocol.

### RNA extraction, PCR and real-time PCR

RNA was extracted using the PureLink RNA kit (Ambion) and cDNA prepared using the High-Capacity cDNA Reverse Transcription Kit (Applied Biosystems). Target gene expression was analyzed by Real-time PCR (qRT-PCR) (supplementary Table 1) and normalized to endogenous 18s expression as previously described [31]. Primers used for PCR are listed in supplementary Table 1.

### RNA stability assay

Actinomycin D (Sigma) treatment (10μg/ml) was performed for the indicated time points as previously reported [31] and RNA was extracted and analyzed by qRT-PCR as described above.

### Human colorectal cancer tumor samples and immunohistochemistry

For immunohistochemical evaluation of ESRP1, five TMA with 1mm cores from colon cancer (80 cases), prepared as previously described, were obtained from the Dept. of Surgical Pathology, University of Turin [21]. After antigen retrieval, sections were stained with anti-ESRP1 antibody (Sigma) and revealed with biotinylated anti-rabbit antibody and the ABC complex (DAKO) followed by exposure to 3, 3′*-Diaminobenzidine* (DAB, Roche).

### Colorectal cancer expression datasets

Gene expression data from the 151 CRC cell lines were extracted from the Gene Expression Omnibus (GEO, accession: GSE59857), normalized and preprocessed (expression filtering, removal of redundant probes, log_2_ transformation) as previously described [23]. ESRP1 expression was analyzed in CRC TCGA samples, using our previously assembled 450-sample TCGA mRNA dataset, as described elsewhere [32].

### Gene expression profiling by cDNA microarray analysis

For gene expression profiling, RNA was extracted using miRNeasy Mini Kit (Qiagen), according to the manufacturer’s protocol. RNA quantification, quality assessment, cRNA synthesis, hybridization and data processing as well as GSEA analysis are described in supplementary methods. ESRP1-overexpressed and ESRP1-silenced Caco-2 cells were compared to Empty and Scr controls, respectively, by applying a double filter based on t-test (P <0.01) and > 1.5 fold-change (absolute log2 ratio).

### Proliferation assay

Cells (2×10^3^/well) were cultured in 96-well plates in monolayer or suspension (ultra-low attachment, Corning) for the indicated time points and performed as previously described [31].

### Soft agar assay

The soft agar assay was performed as described elsewhere [51]. Briefly, 2×10^5^ Caco-2 cells and 1×10^4^ HCA24, COLO320DM, COGA8, HDC142 cells o 2.4×10^4^ Snu-C1 cells were plated in the upper layer and stained 2 months or 3-4 weeks later, respectively, with p-nitroblue tetrazolium (NBT, Sigma) for colony visualization and counting.

### Western blotting

Protein was extracted from cells or from OCT-embedded normal colon sections as described in supplementary methods and separated by 4-15% SDS-PAGE (Biorad). Antibodies used are described in supplementary Table 2. Densitometric analysis was performed using the volume analysis tool of ImageLab software (Biorad Laboratories Inc).

### ELISA

Caco-2 cells (1×10^6^) were grown in 3ml serum-free media in 6cm ultra-low attachment and human FGF7 was measured using the Human FGF-7 ELISA Kit (Sigma) according to the manufacturer’s instructions.

### RNA-Immunoprecipitation (RNA-IP)

Cell protein extracts were prepared and used for immunoprecipitation with anti-ESRP1 antibody or rabbit IgG and for RNA extraction as we previously described [31].

### Rescue experiments

For rescue experiments, site directed mutagenesis was perfomed on human ESRP1 cDNA in pLX304 using QuikChange Lightning Site-directed mutagenesis kit (agilent technologies) as per manufacturer's protocol. Primers used are described in Supplementary Table 1. Reverse transfection with lipofectamine 2000 was used for delivery plasmid DNA into cells (Invitrogen).

### Primary tumor generation, experimental metastasis and ethics statement

All animal procedures were in accordance with Italian legislation on animal experimentation, and were approved by the Animal Care Committee of the Candiolo Cancer Institute and by the Italian Ministry of Health. For primary tumor generation, Caco-2 cells (2.5 × 10^6^) were subcutaneously injected in NOD-SCID-IL2Rg-null (NSG) mice. When tumors reached ∼1cm in diameter in control or ESRP1-modulated condition, mice were sacrificed by carbon dioxide inhalation followed by cervical dislocation. For experimental metastasis, Caco-2 (2.5 × 10^6^) or COLO320DM (1 × 10^5^) cells were intravenously injected in NSG mice and macrometastases were followed weekly by magnetic resonance imaging (MRI) with an Aspect M2 System (Aspect Magnet Technologies Ltd., Netanya, Israel) working at 1 Tesla by using a dedicated bed for immunocompromised mice to maintain sterile conditions. T2-weighted (T_2w_) anatomical images were acquired with a fast spin echo sequence (TR 3300 s; TE effective 44 ms; number of slices 25; slice thickness 1.0 mm; FOV 100 mm; acquisition matrix 256 × 256; two averages) as previously described [52].

### Statistical analyses

Data are expressed as mean ± standard error of mean (s.e.m) or standard deviation (s.d). Statistical differences were determined by a 2-tailed Student’s *t* -test (* *P* <0.05, ** *P* <0.01, *** *P* <0.001). Error bars in all figures denote s.e.m.

## SUPPLEMENTARY DATA






