# Supplementary material for: The RNA-binding protein ESRP1 promotes human colorectal cancer progression
Source: Oncotarget. 2016 Dec 28;8(6):10007–24. doi: 10.18632/oncotarget.14318 (PMC5354637; doi:10.18632/oncotarget.14318)
Supplement: Supplementary file 1 [file oncotarget-08-10007-s001.pdf]

The RNA-binding protein ESRP1 promotes human colorectal cancer progression

SUPPLEMENTARY DATA

CRC cell culture

CRC cells used in this study were cultured in the following media: Caco-2cells in DMEM (Invitrogen), 20% FBS, non- essential amino acids (Invitrogen), sodium pyruvate (Invitrogen) and 100μg/ml penicillin/streptomycin (PS, Invitrogen); COLO320DM cells in RPMI (Invitrogen), 10% FBS and PS; HCA24 in DMEM, 10% FBS and PS; HCA24 cells in DMEM, 10% FBS and PS.

cDNA microarray analysis

The quantification and quality analysis of RNA was performed on a Bioanalyzer 2100 (Agilent), using RNA 6000 nano Kit (Agilent). Synthesis of cDNA and biotinylated cRNAwas performed using the IlluminaTotalPrep RNA Amplification Kit (Ambion), according to the manufacturer's protocol using 500 ng of total RNA. Quality assessment and quantification of cRNAswere performed with Agilent RNA kits on Bioanalyzer 2100. Hybridization of cRNAs (750 ng) was carried out using Illumina Human 48 k gene chips (Human HT-12 V4 BeadChip). Array washing was performed using Illumina High Temp Wash Buffer for 10 mins at 55 °C, followed by staining using streptavidin-Cy3 dyes (Amersham Biosciences). Probe intensity data were obtained and normalized using the Illumina Genome Studio software (Genome Studio V2011.1) and further processed with Excel software. Subsequent data processing, carried out with Excel (Microsoft), included: (i) scaling, Log_2 transformation and detection filtering; (ii) removal of redundant probes (>average); (iii) Log_2Ratio transformation and selection of genes differentially expressed; Log_2Ratio expression data were clustered and visualized using the GEDAS software [1] .

GSEA analysis and ingenuity pathway analysis

GSEA software was downloaded from the Broad Institute GSEA portal http://software.broadinstitute.org/gsea/index.jsp) and applied, using log_2ratio for gene ranking. The significance of enrichment was estimated using 1000 gene permutations. For Ingenuity Pathway analysis, Genes Symbol and log2ratio values of each gene selected in the ESRP1 signature were uploaded on IPA http://www.ingenuity.com. Standard Core analysis was performed.

Classification of ESRP1 expression according to CRC subtypes

TCGA gene expression data of COADREAD samples were downloaded from UCSC cancer browser [2]. COADREAD primary solid tumor samples (n=380) were subdivided in 6 different subtypes based on their gene expression profiles, according to Marisa L. et al, 2013[3], by means of 'citccmst'[4] R [5] package. Three hundred COADREAD samples were univocally classified as belonging to a specific subtype. Statistical significance of difference in ESRP1 expression level in the different subtypes was calculated by a *post-hoc* analysis with Tukey's HSD test in conjunction with ANOVA test.

Protein extraction

Protein was extracted from CRC cells using TENT buffer (50mM Tris-HCl, 5mM EDTA, 150 mMNaCl, 1% Triton-X100) supplemented with protease inhibitors (Complete Mini, Roche) and a cocktail of phosphatase inhibitors (5mM sodium fluoride, 1mM sodium vanadate and 1mM PMSF). Proteinwas extracted from OCT-embedded sections using lysis buffer containing 50mM Tris-HCl, 137.5mM sodium chloride, 10% glycerol, 0.5% SDS, 5mM EDTA and inhibitor of proteases.

REFERENCES

1. Warzecha CC, Sato TK, Nabet B, et al. ESRP1 and ESRP2 are epithelial cell-type-specific regulators of FGFR2 splicing. Molecular cell 2009;33:591-601.
2. Zhang H, Hylander BL, LeVea C, et al. Enhanced FGFR signalling predisposes pancreatic cancer to the effect of a potent FGFR inhibitor in preclinical models. British journal of cancer 2014;110:320-9.

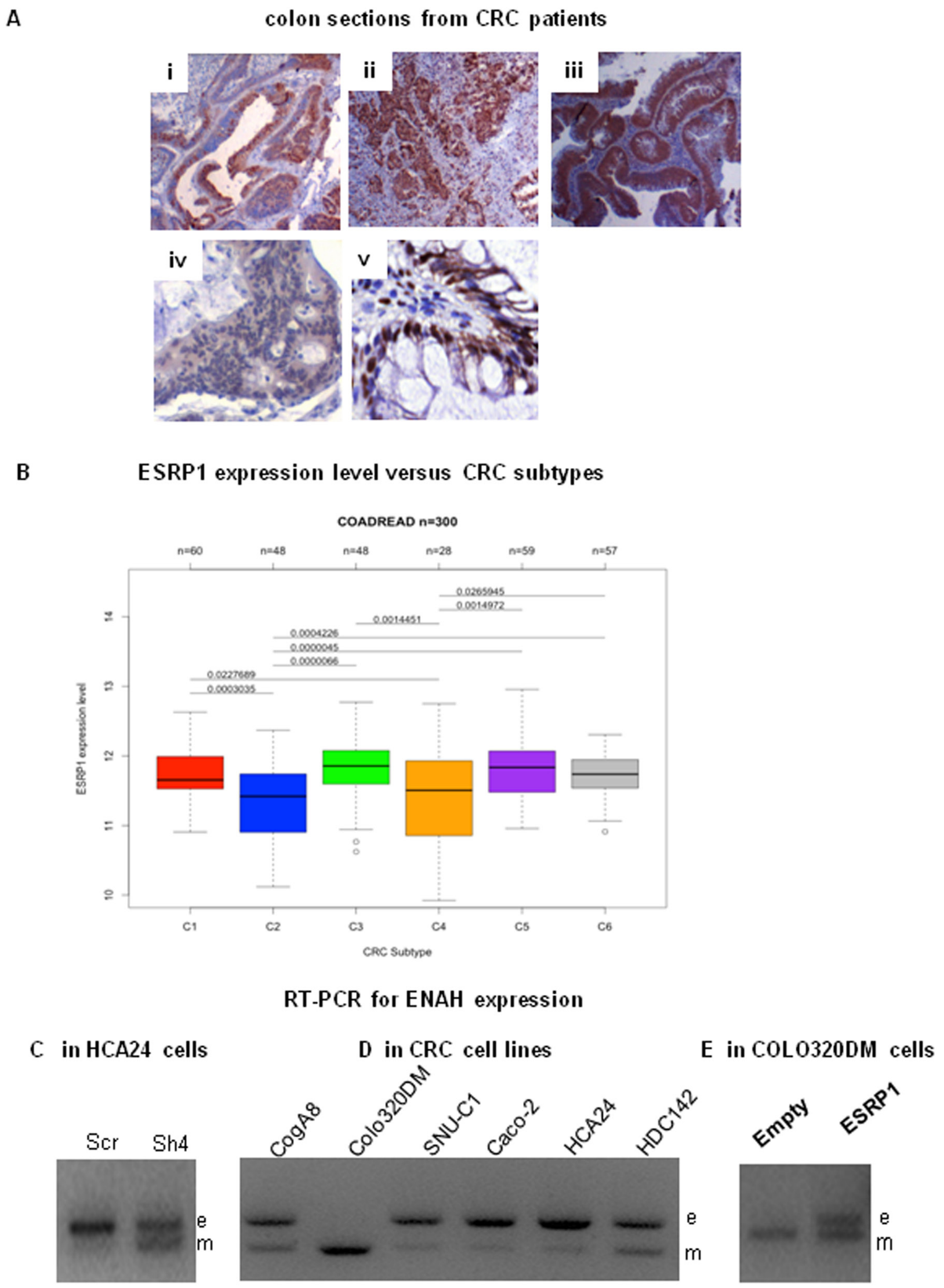

Supplementary Figure 1: Analysis of ESRP1 expression and ESRP1-regulated gene expression in human CRC samples and cell lines. A. Immunohistochemical analysis of ESRP1 expression in sections from human CRC tumors (i-iv) and normal colon crypt (v), Scale bar =100µm. **B.** ESRP1 expression extracted from different CRC subtypes [22]. RT-PCR analysis of ENAH alternatively spliced isoforms (e-epithelial; m-mesenchymal) **C.** in ESRP1-silenced and control HCA24 cells, **D.** in CRC cell lines used in this study, and **E.** in ESRP1-overexpressing and control COLO320DM cells.

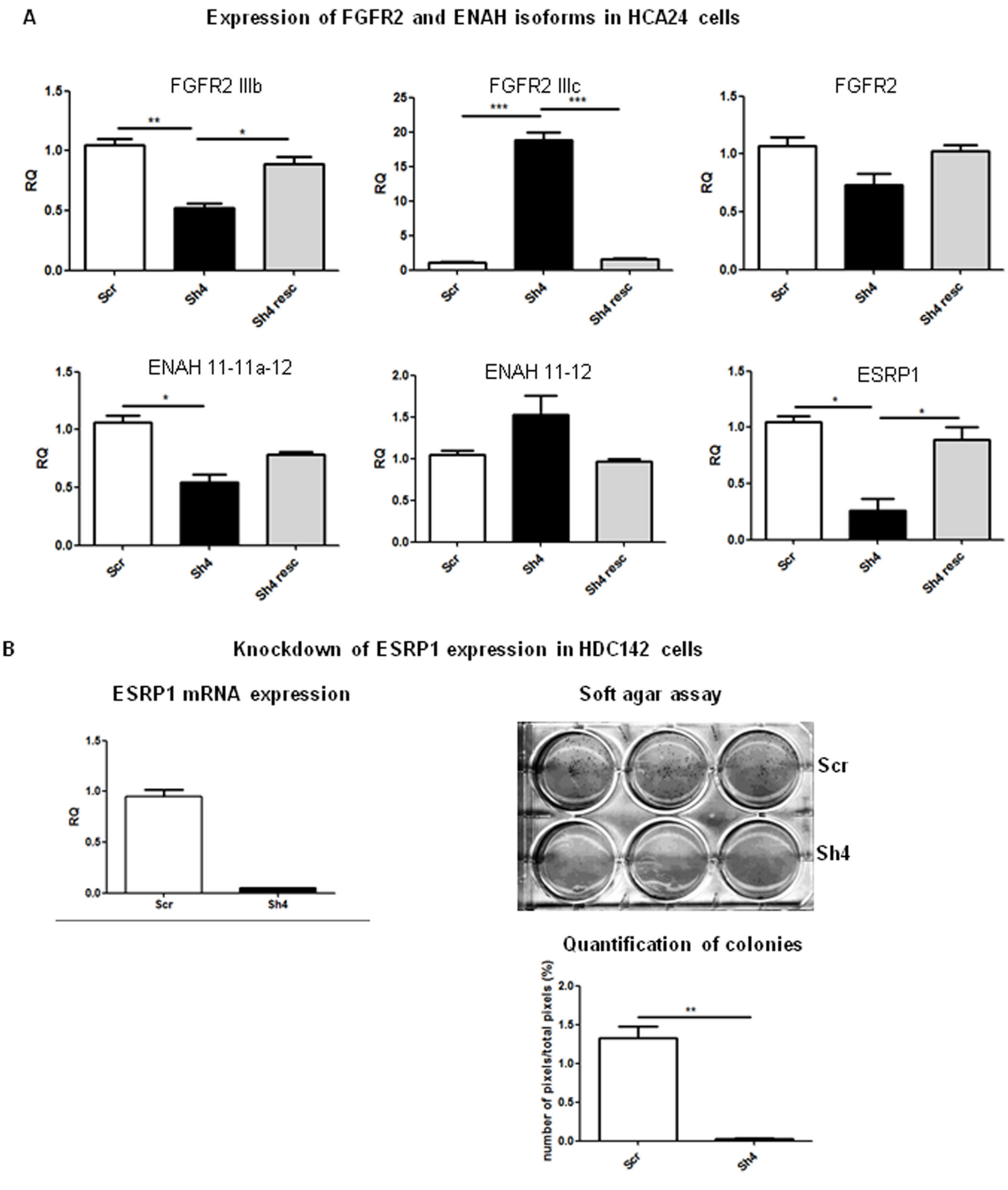

Supplementary Figure 2: Analysis of ESRP1-regulated gene expression in HCA24 cells and effect of ESRP1-silencing on HDC142 cells. A. qRTPCR analysis of ESRP1-regulated gene expression in HCA24 cells. **B.** qRT-PCR analysis of ESRP1 expression in ESRP1-silenced (Sh4) and control HDC142 cells (n=4, 2 independent experiments). Soft agar assay with ESRP1-silenced and control HDC142 cells (n=3, 2 independent experiments) and Image J software quantification of pixels/well (n=6).

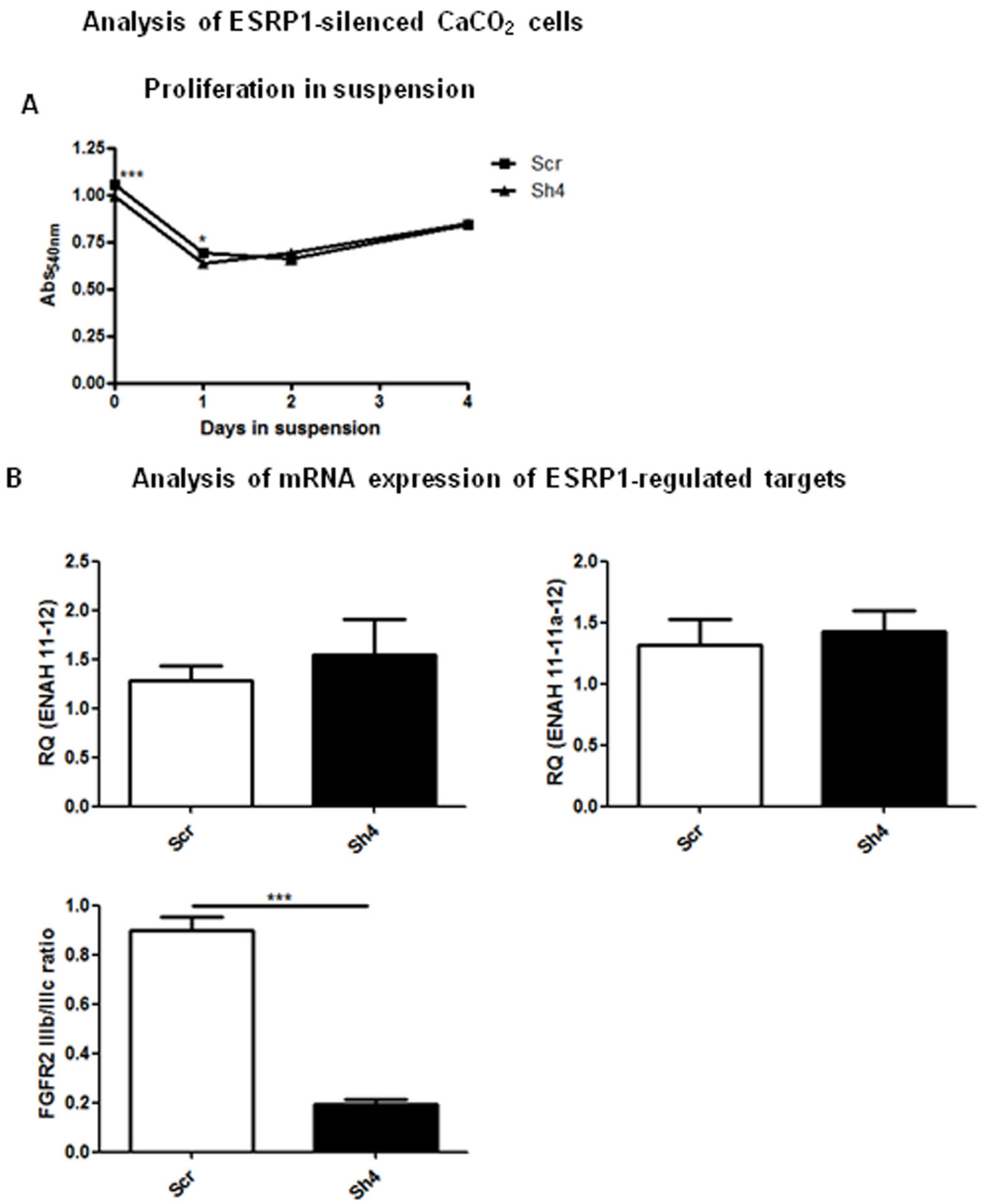

Supplementary Figure 3: Analysis of effect of ESRP1-silencing in Caco-2 cells. A. MTT assay showing proliferation of ESRP1-silenced and control Caco-2 cells grown in suspension for 4 days (n=8). **B.** qRTPCR analysis of ESRP1-regulated gene expression in ESRP1-silenced Caco-2 cells (n=6, 2 independent experiments).

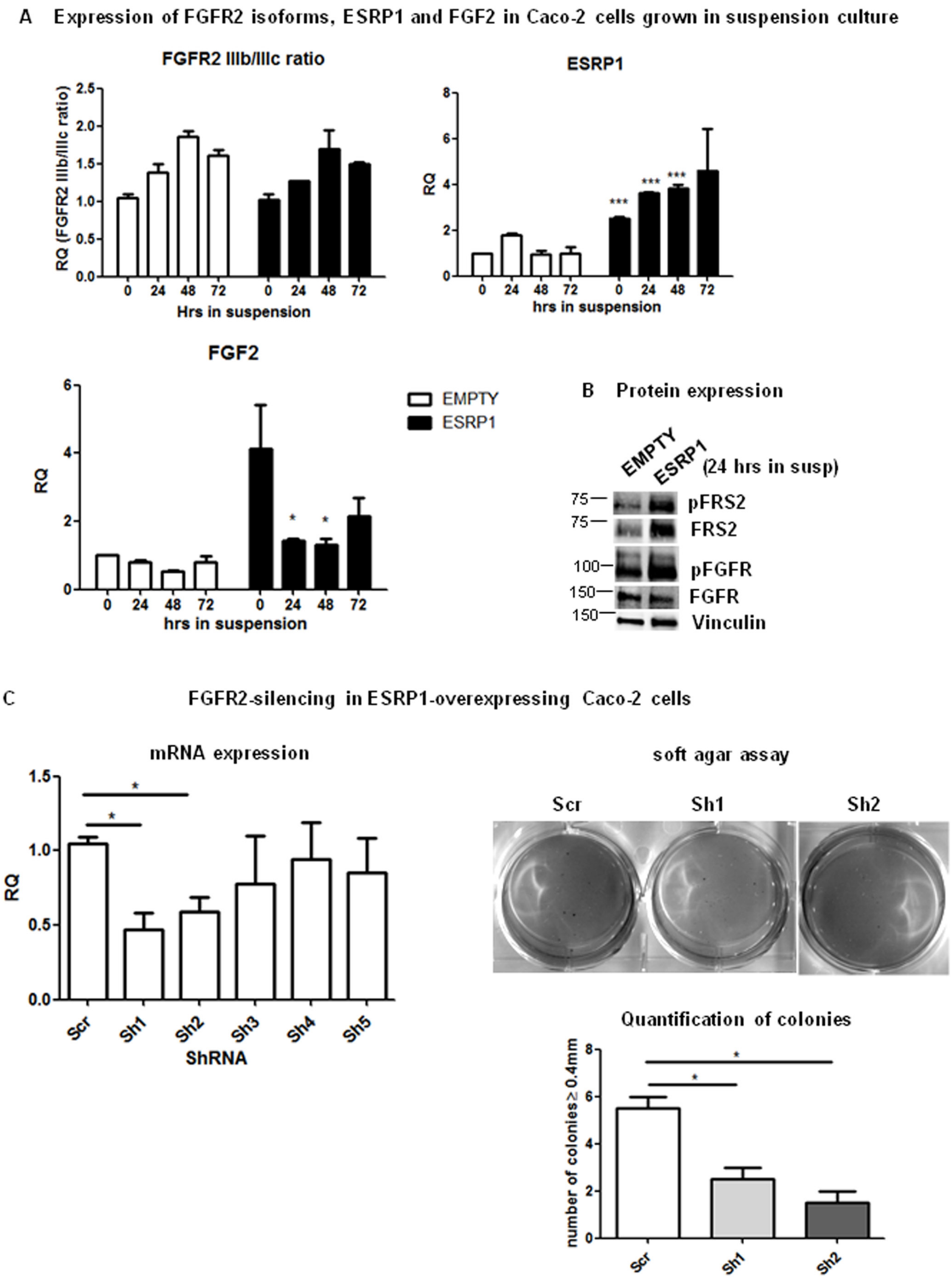

Supplementary Figure 4: Analysis of FGFR pathway in Caco-2 cells grown in suspension and anchorage-independency. **A.** qRT-PCR analysis of the expression of epithelial and mesenchymal FGFR2 isoforms, as well as of ESRP1, and FGF2 is shown in ESRP1-overexpressing and control Caco-2 cells grown in suspension for 72 h (n=4). **B.** Western blot analysis of FRS2 expression in ESRP1-overexpressing Caco-2 cells grown in suspension for 24h compared to untreated Empty controls. **C.** qRTPCR analysis of FGFR2 expression in ShRNA-mediated FGFR2 silencing in ESRP1-overexpressing Caco-2 cells. Soft agar assay of ESRP1-overexpressing Caco-2 cells silenced for FGFR2 (Sh1 and Sh2) *versus* Scr controls (n=3, 2 independent experiments). Quantification of colonies ≥4mm is included.

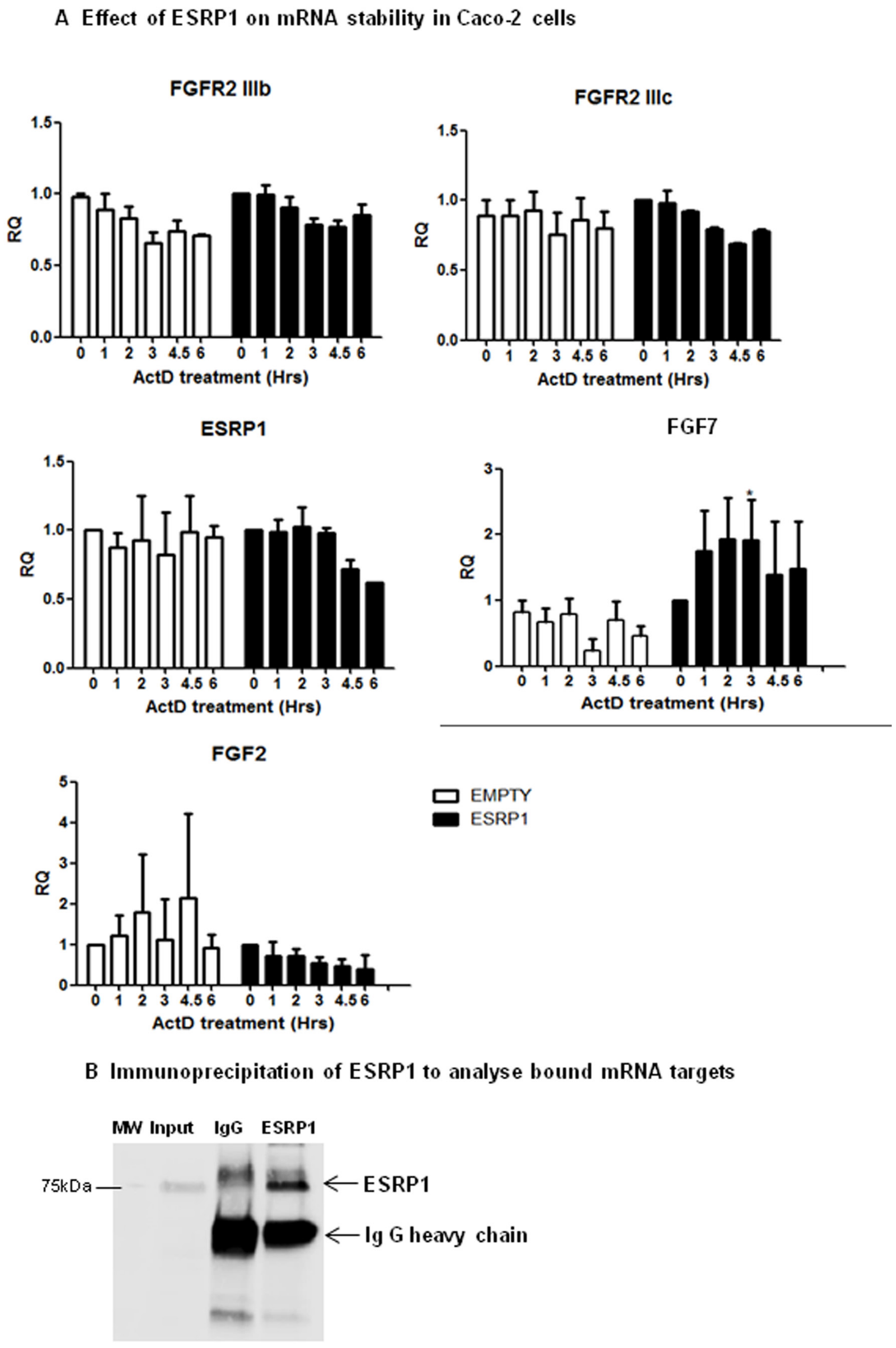

Supplementary Figure 5: Effect of ESRP1 on mRNA stability in Caco-2 cells and Immunoprecipitation of ESRP1 to analyse bound mRNA targets. A. qRT-PCR analysis of mRNA stability of FGFR2 isoforms, ESRP1, FGF7 and FGF2 after actinomycin D treatment in ESRP1-overexpressing and control Caco-2 cells grown in suspension for 72 h (n=4, 2 independent experiments). **B.** Western blot analysis of ESRP1 immunoprecipitation. Rabbit IgG was used as negative control.

Rare micrometastasis generated by ESRP1-overexpressing Caco-2 cells

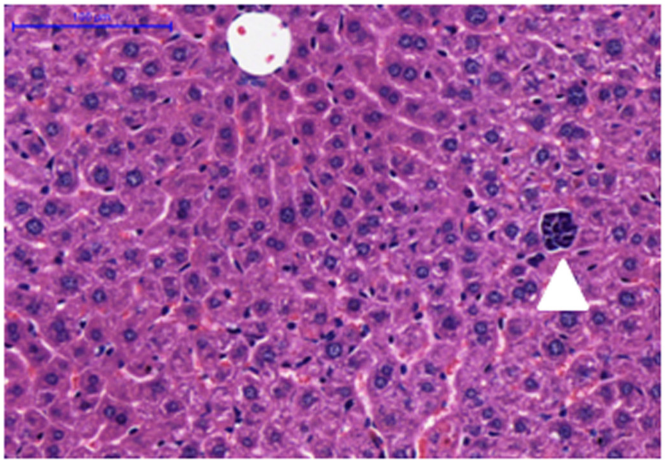

Supplementary Figure 6: Analysis of NSG mice livers after injection of ESRP1-overexpressing Caco-2 cells. Micrometastasis (arrowhead) in the hematoxylin/eosin-stained liver section is shown.

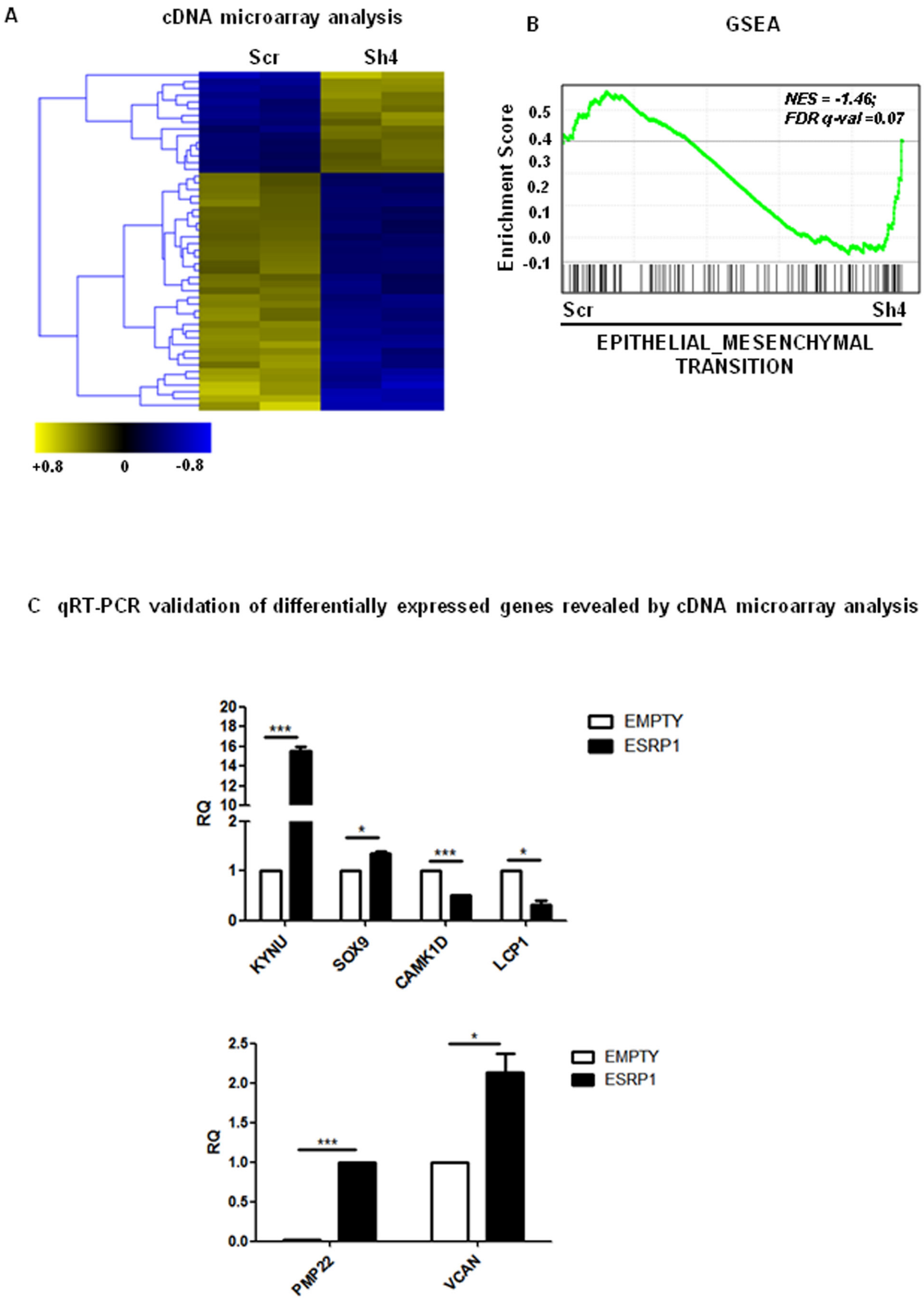

Supplementary Figure 7: Effects of ESRP1-silencing on Caco-2 cells. A. cDNA microarray analysis of ESRP1-silenced and control Caco-2 cells under basal conditions. The heatmap reports the expression (log_2 ratio against average) of 50 genes differentially modulated after ESRP1 silencing in Caco-2 cells. **B.** GSEA of the transcription profiles for selected enrichment term. **C.** qRT-PCR validation of genes differentially expressed between ESRP1-overexpressing Caco-2 cells versus Empty controls.

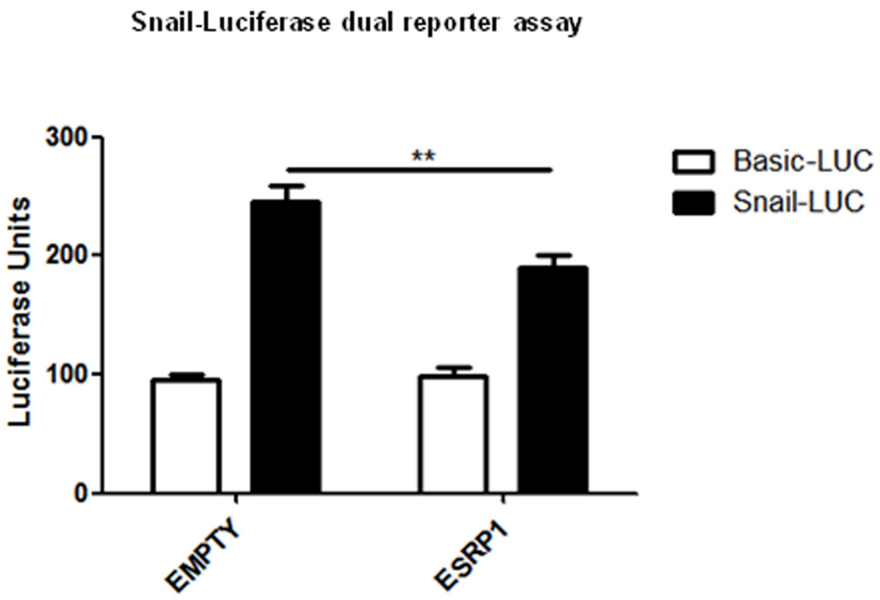

Supplementary Figure 8: Snail-Luciferase dual reporter assay. Snail activity in ESRP1-overexpressing Caco-2 cells *versus* Empty controls under basal conditions (adherent cells) are shown.

Supplementary Table 1: Primers used in this study

RT-PCR primers		
**Primer name**	**Sequence (5’-3’)**	**Reference**
ENAH	TGCTGGCCAGGAGGAGAAGAAT<br>ACTGGGCTGTGATAAGGGTGTGG	1
**qRT-PCR primers (Roche UPL)**		
**Primer name #UPL**	**Sequence (5’-3’)**	
ESRP1 #37	TGATCTTCGAAAAGAATTCAAGAAA<br>CGAGAGACTGAACTACTCTTCTCAAAA	
ESRP1 #52	CCCACCGCCATGTAAGTT<br>GCAGGAGCTGGAAATGTGTAG	
E-cadherin (E-cad)#35	CCCGGGACAACGTTTATTAC<br>GCTGGCTCAAGTCAAAGTCC	
Vimentin (Vim)#13	TACAGGAAGCTGCTGGAAGG<br>ACCAGAGGGAGTGAATCCAG	
FGF7 #59	AAGGGACCCAAGAGATGAAGA<br>CCTTTGATTGCCACAATTCC	
FGF2 #7	TTCTTCCTGCGCATCCAC<br>CCTCTCTCTTCTGCTTGAAGTTG	
Snail #11	GCTGCAGGACTCTAATCCAGA<br>ATCTCCGGAGGTGGGATG	
KYNU#68	GCCTGCTGGTGTTCCTACA<br>AATGAAGGCACCAGCAATTC	
LCP1 #37	AACCCTCGAGTCAATCATTTG<br>TTTGATCTTTTCATAGAGCTGGAA	
CAMK1D#27	TGGAGAAGGACCCGAATAAA<br>TGTGTCACCAGCGATCCA	
SOX9#61	GTACCCGCACTTGCACAAC<br>TCTCGCTCTCGTTCAGAAGTC	
VCAN#9	TCCCCAGGAAACTTACGATG<br>GGGGACAGTGAGGTGGAA	
PMP22#63	AGAAGGGGTTACGCTGTTTG<br>GGAGGACGATGATACTCAGCA	
**qRT-PCR primers (SYBR)**		
**Primer name**	**Sequence (5’-3’)**	**Reference**
FGFR2 IIIb	GATAAATAGTTCCAATGCAGAAGTGCT<br>TGCCCTATATAATTGGAGACCTTACA	2
FGFR2 IIIc	GGATATCCTTTCACTCTGCATGGT<br>TGGAGTAAATGGCTATCTCCAGGTA	2
ENAH 11-12	GGACAAAGGTGAAGATTCAGAGCCTG<br>GGGTGTGGATTTTGGTCTGGAGATAAC	
ENAH 11a Rev	CTGATTTTTCCTTGGAGAATCCCG	
**ESRP1 mutagenesis primer**		
**Primer name**	**Sequence (5’-3’)**	
hESRP1mut_Fw	GAGAAGAGTAGTGCAGTCTCGCGATATGGAGCCTCTC	

Roche Universal probe libraries (UPL) were used for qRT-PCR.

Supplementary Table 2: Antibodies used in this study

Antibody	Company
ESRP1	Sigma-Aldrich
pERK	Cell signaling Technology
ERK	Santa Cruz Biotechnology
$pAKT^{ser473}$	Cell signaling Technology
AKT	Cell signaling Technology
Snail	Santa Cruz Biotechnology
Twist	Santa Cruz Biotechnology
pFGFR	Cell signaling Technology
FGFR1	Cell signaling Technology
FGFR2	Cell signaling Technology
pFRS2	Cell signaling Technology
FRS2	Santa Cruz Biotechnology
Vinculin	In-house
Actin	Santa Cruz Biotechnology

Supplementary Table 3: Table listing Illumina probe ID, gene symbol and fold change (log2 Ratio) of the genes differentially expressed in Caco-2 cells upon ESRP1 overexpression or silencing

See Supplementary File 1

Supplementary Table 4: List of modulated EMT signature genes (highlighted in yellow) following ESRP1 overexpression in Caco-2 cells

See Supplementary File 2
