# Supplementary material for: The RNA-binding protein ESRP1 promotes human colorectal cancer progression
Source: Oncotarget. 2016 Dec 28;8(6):10007–24. doi: 10.18632/oncotarget.14318 (PMC5354637; doi:10.18632/oncotarget.14318)
Supplement: Supplementary file 3 [file oncotarget-08-10007-s003.docx]

Table 4: EMT signature genes significantly upregulated following ESRP1 overexpression in Caco-2 cells

| **DEFINITION** | **Probe ID** | **CACO2 EMPTY (L2S)** | **CACO2 ESRP1+ (L2S)** | **CACO2 ESRP1+/Empty L2R** | **Selected in**  **ESRP1+**  **signature** |
| --- | --- | --- | --- | --- | --- |
| Homo sapiens peripheral myelin protein 22 (PMP22), transcript variant 2, mRNA. | 7560138 | 9,5944 | 11,57769 | 1,983285 | 1 |
| Homo sapiens versican (VCAN), mRNA. | 5910113 | 8,59485 | 10,46363 | 1,86879 | 1 |
| Homo sapiens matrix metallopeptidase 1 (interstitial collagenase) (MMP1), mRNA. | 3360224 | 9,81684 | 11,26082 | 1,44398 | 1 |
| Homo sapiens insulin-like growth factor binding protein 4 (IGFBP4), mRNA. | 7510414 | 8,91746 | 10,21066 | 1,293196 | 0 |
| Homo sapiens serpin peptidase inhibitor, clade E (nexin, plasminogen activator inhibitor type 1), member 2 (SERPINE2), mRNA. | 5080192 | 8,39756 | 9,581069 | 1,183513 | 0 |
| Homo sapiens procollagen C-endopeptidase enhancer 2 (PCOLCE2), mRNA. | 4810445 | 7,59195 | 8,678363 | 1,086415 | 0 |
| Homo sapiens cadherin 6, type 2, K-cadherin (fetal kidney) (CDH6), mRNA. | 3800241 | 8,29885 | 9,364695 | 1,065847 | 1 |
| Homo sapiens TIMP metallopeptidase inhibitor 1 (TIMP1), mRNA. | 7650433 | 8,4615 | 9,450716 | 0,989215 | 0 |
| Homo sapiens laminin, alpha 3 (LAMA3), transcript variant 1, mRNA. | 6480592 | 8,3083 | 9,293138 | 0,984834 | 1 |
| Homo sapiens laminin, alpha 1 (LAMA1), mRNA. | 2190113 | 9,61432 | 10,51465 | 0,900336 | 0 |
| Homo sapiens transforming growth factor, beta receptor III (TGFBR3), mRNA. | 3190379 | 10,4114 | 11,21413 | 0,802737 | 0 |
| Homo sapiens gap junction protein, alpha 1, 43kDa (GJA1), mRNA. | 6940017 | 10,0993 | 10,87068 | 0,771399 | 0 |
| Homo sapiens vimentin (VIM), mRNA. | 830593 | 10,2661 | 11,01248 | 0,746367 | 0 |
| Homo sapiens cysteine-rich, angiogenic inducer, 61 (CYR61), mRNA. | 3930605 | 10,5699 | 11,2872 | 0,717353 | 0 |
| Homo sapiens collagen, type XII, alpha 1 (COL12A1), transcript variant short, mRNA. | 3060095 | 9,27462 | 9,989419 | 0,714801 | 0 |
| Homo sapiens fibromodulin (FMOD), mRNA. | 1710484 | 8,93241 | 9,613962 | 0,681553 | 0 |
| Homo sapiens collagen triple helix repeat containing 1 (CTHRC1), mRNA. | 4860546 | 7,97459 | 8,602972 | 0,628379 | 0 |
| Homo sapiens integrin, alpha 2 (CD49B, alpha 2 subunit of VLA-2 receptor) (ITGA2), mRNA. | 3890541 | 9,9208 | 10,53356 | 0,61276 | 0 |
| Homo sapiens CD59 molecule, complement regulatory protein (CD59), transcript variant 2, mRNA. | 4760593 | 9,38153 | 9,978164 | 0,596632 | 0 |
| Homo sapiens spermidine/spermine N1-acetyltransferase 1 (SAT1), mRNA. | 5490431 | 13,1986 | 13,78449 | 0,585878 | 1 |
